# Test-retest variability of left ventricular 4D flow cardiovascular magnetic resonance measurements in healthy subjects

**DOI:** 10.1186/s12968-018-0432-4

**Published:** 2018-03-02

**Authors:** Victoria M. Stoll, Margaret Loudon, Jonatan Eriksson, Malenka M. Bissell, Petter Dyverfeldt, Tino Ebbers, Saul G. Myerson, Stefan Neubauer, Carl- Johan Carlhäll, Aaron T. Hess

**Affiliations:** 1 University of Oxford Centre for Clinical Magnetic Resonance Research (OCMR), Division of Cardiovascular Medicine, Radcliffe Department of Medicine, Oxford, UK; 2 Division of Cardiovascular Medicine, Linköping University, Linköping, Sweden

**Keywords:** Left ventricular, 4D flow, Repeatability, Variability, Kinetic energy

## Abstract

**Background:**

Quantification and visualisation of left ventricular (LV) blood flow is afforded by three-dimensional, time resolved phase contrast cardiovascular magnetic resonance (CMR 4D flow). However, few data exist upon the repeatability and variability of these parameters in a healthy population. We aimed to assess the repeatability and variability over time of LV 4D CMR flow measurements.

**Methods:**

Forty five controls underwent CMR 4D flow data acquisition. Of these, 10 underwent a second scan within the same visit (scan-rescan), 25 returned for a second visit (interval scan; median interval 52 days, IQR 28–57 days). The LV-end diastolic volume (EDV) was divided into four flow components: 1) Direct flow: inflow that passes directly to ejection; 2) Retained inflow: inflow that enters and resides within the LV; 3) Delayed ejection flow: starts within the LV and is ejected and 4) Residual volume: blood that resides within the LV for > 2 cardiac cycles. Each flow components’ volume was related to the EDV (volume-ratio). The kinetic energy at end-diastole (ED) was measured and divided by the components’ volume.

**Results:**

The dominant flow component in all 45 controls was the direct flow (volume ratio 38 ± 4%) followed by the residual volume (30 ± 4%), then delayed ejection flow (16 ± 3%) and retained inflow (16 ± 4%). The kinetic energy at ED for each component was direct flow (7.8 ± 3.0 microJ/ml), retained inflow (4.1 ± 2.0 microJ/ml), delayed ejection flow (6.3 ± 2.3 microJ/ml) and the residual volume (1.2 ± 0.5 microJ/ml). The coefficients of variation for the scan-rescan ranged from 2.5%–9.2% for the flow components’ volume ratio and between 13.5%–17.7% for the kinetic energy. The interval scan results showed higher coefficients of variation with values from 6.2–16.1% for the flow components’ volume ratio and 16.9–29.0% for the kinetic energy of the flow components.

**Conclusion:**

LV flow components’ volume and their associated kinetic energy values are repeatable and stable within a population over time. However, the variability of these measurements in individuals over time is greater than can be attributed to sources of error in the data acquisition and analysis, suggesting that additional physiological factors may influence LV flow measurements.

**Electronic supplementary material:**

The online version of this article (10.1186/s12968-018-0432-4) contains supplementary material, which is available to authorized users.

## Background

The main purpose of the cardiovascular system is to drive, control and maintain blood flow through the heart and vessels [1]. Insights into intra-cardiac blood flow are now afforded by the use of retrospectively electrocardiogram (ECG) gated, three-dimensional (3D), time resolved flow encoded cardiovascular magnetic resonance (CMR) (3D + time = 4D flow) [2–6]. The 4D flow within the left ventricle (LV) can be separated into four functional flow components and the kinetic energy (KE) of the blood throughout the cardiac cycle can be quantified [3, 7, 8]. In healthy hearts these functional flow components have specific routes and energetics that may represent important aspects of normal ventricular function [9]. Typically a third of the inflow to the LV passes directly through to the aorta in healthy hearts allowing a preservation of LV inflow KE, which may assist with an efficient systolic ejection phase. Alterations in LV blood flow components have been found in patients with early compensated dilated cardiomyopathy, where a substantial proportion of the inflow is retained within the LV and there is an associated decrease in preservation of the LV inflow KE [8]. These findings suggest that the volume and KE of the 4D flow components may be sensitive biomarkers for the early detection of cardiac pathology. 4D flow also provides a potential future tool for the evaluation of therapeutic interventions [10]. However the use of 4D flow components for early diagnosis and monitoring of changes in individual patients requires an understanding of the intra-subject repeatability of the measures and the variability of these parameters over time.

To date, studies reporting healthy control data have enrolled small numbers, typically 6–17 participants with data acquired at a single time point [3–5, 8] and none have assessed the stability of LV 4D flow components over time. Thus, this study aims to understand the stability of the volume and KE profiles of LV flow components in healthy participants. In order to achieve this we first assessed the repeatability of the 4D flow data acquisition, post-processing and analysis in order to understand the error associated with the technique. Subsequently we determined how these components change over time by repeating a data acquisition after a period of a few weeks.

## Methods

### Study population

Forty five healthy subjects were prospectively recruited specifically for the aims of this study. All participants had no contraindication to CMR scanning, no history of cardiac disease, nor symptoms of cardiac disease. This study was approved by the local research ethics committee and written informed consent was obtained from each participant.

Ten of the participants underwent two 4D flow data acquisitions within the same study visit to assess ‘scan-rescan’ repeatability. Between each data acquisition the participant was removed completely from the scanner so each data set was acquired with the same potential real-life sources of variance, including subtle changes in subject positioning in the CMR system. Twenty five of the participants returned for a second ‘interval’ 4D flow data acquisition at least 10 days later (52 ± IQR 28–57 days). The participants in the scan-rescan and interval groups were different as this study was conducted in two phases. Additional file 1: Table S1 shows that the two groups were similar for cardiac function measurements.

### Anthropometric measurements

Height and weight were recorded and body mass index (BMI) calculated. Blood pressure was recorded as an average of 3 supine measurements taken over 10 min (DINAMAP-1846-SX, Critikon Corporation; General Electric Healthcare, Waukesha, Wisconsin, USA). Heart rate was recorded at the time of the short axis stack acquisition.

### Cardiovascular magnetic resonance protocol

Each participant underwent CMR imaging on a 3 Tesla system (Trio, Siemens Healthineers Erlangen, Germany) using a 32 channel cardiac coil. All images were ECG-gated. Images for LV volumes were acquired using retrospectively gated balanced steady-state free precession (bSSFP) cine sequences scan parameters were echo time 1.5 ms, repetition time 3 ms and flip angle 50°. Slice thickness was 8 mm with contiguous slice position for the short axis stack. Each cine slice was acquired during a single breath hold, as a free breathing method was not available, this protocol allowed shorter breath holds (which will be helpful when this technique is utilised in patients), and easier repetition if any mis-triggering or breathing artefact occurred during data acquisition. Cine images were analysed using cmr42 (Circle Cardiovascular Imaging Inc., Calgary, Canada) as previously described [11].

4D flow data acquisitions were acquired during free breathing, using a retrospectively ECG triggered, respiratory navigator gated three dimensional (3D), three directional, time resolved phase contrast CMR sequence with data measured over many cardiac cycles. The echo time was 2.75 ms with a repetition time of 4.3 ms and temporal resolution of 52 ms. The flip angle was 7°, read field of view 390 mm and voxel size 3x3x3 mm^3^. The velocity encoding was 100 cm/s. The field-of-view (FOV) was sagittal and adjusted for each subject to fully encompass the whole heart. The data acquisition times were between 15 and 20 min. While the data are presented as a single cardiac cycle they capture the complete cardiac cycle which means they can be used to form a closed loop where the first and last frame are also consecutive frames. For illustration: if the 4D flow loop is concatenated after itself (doubling the number of time frames) the data will appear continuous and to have captured two cardiac cycles.

### Post processing and data analysis

Background phase offsets were corrected with a third-order polynomial fit. Data quality control steps were applied as previously described [3] using automated customised Matlab software (The Mathworks Inc., Natick, Massachusetts, USA). Velocity data was converted into a file format compatible with commercially available visualisation software (EnSight, CEI Inc., Research Triangle Park, North Carolina, USA).

**Fig. 1:**
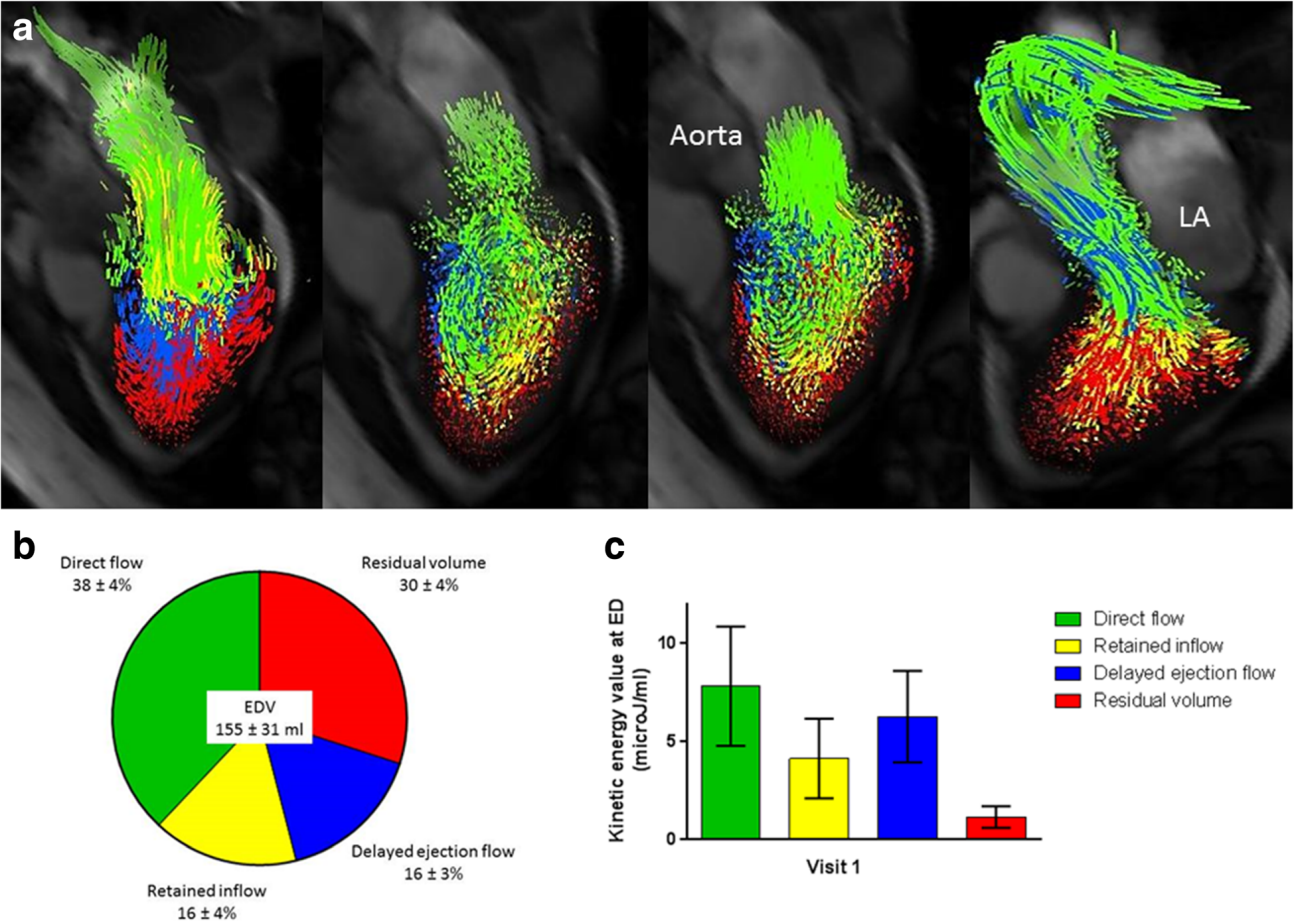
Visualisation and quantification of LV blood flow components’ volume and kinetic energy values at end-diastole for all 45 participants. **a** Flow visualisation throughout the cardiac cycle from left to right panel; early diastole, diastasis, atrial contraction and systolic ejection. LA, left atrium. **b** Flow components by percentage of EDV. **c** Kinetic energy at end-diastole related to blood volume of the 4 flow components

All data sets were analysed using a method previously described by Eriksson et al. [3, 5]. The LV endocardium was manually segmented from short-axis images at end diastole (ED) and end systole (ES), using freely available software (Segment, version 1.9 R2842) [12]. The ED and ES timeframes were determined by visual inspection of the open or closed positions of the aortic and mitral valves and the LV size in the long and short axis images. The segmentation at ED is resampled to give a volume with isotropic voxels equal to the size of the flow data voxels. A pathline is emitted from the centre of each voxel included in the LV segmentation. Pathlines are created forwards and backwards in time until the preceding or subsequent ES, respectively. A pathline is a probabilistic path a finite volume of blood takes through space as a function of time. Combined these forward and backward pathlines represent the entire LV end diastolic volume (EDV) tracked over one complete cardiac cycle. The positions of all pathlines at the time of ES relative to the LV cavity, as defined by the segmentation at ES, are then used to divide them into four functional flow components: direct flow, retained inflow, delayed ejection flow and residual volume as described previously [3, 5, 8]. Direct flow is defined as blood that enters and exits the LV in the analysed cardiac cycle, retained inflow enters the LV but does not exit during the same cardiac cycle, whilst delayed ejection flow starts within the LV but exits during the analysed cardiac cycle and residual volume is the component that resides within the LV for at least 2 cardiac cycles, these are illustrated in Fig. 1a. Accuracy of this quantification was evaluated by comparing the LV inflow components (direct flow and retained inflow) to the LV outflow components (direct flow and delayed ejection flow), any data sets with > 10% difference would have been excluded from further analysis for quality control, however no datasets met this criteria so all acquired datasets were included in the further analysis.

The KE of these flow components can be calculated throughout the cardiac cycle by utilising KE = ½▪ρ_blood_▪V_pathline_▪v^2^_pathline,_ where ρ_blood_ is blood density, V _pathline_ the volume of blood that a pathline is emitted from, equal to one voxel, and v _pathline_ the velocity of the pathline at a given time point. The KE for each component is the sum of all pathlines in the group. The KE values were calculated over the cardiac cycle and reported at ED, as the KE values at this time-point reflect the preservation of the inflowing KE prior to the rapid ejection of blood during systole. The KE at ED for each component was then divided by the components volume to give a KE per millilitre value, therefore removing any variation due to LV cavity size.

### Intra- and inter-observer variability

Intra-observer variability was determined by an operator experienced in CMR who conducted two blinded assessments of 10 randomly selected data sets, with each assessment separated by more than one month. Inter-observer variability was conducted independently by a second observer experienced in CMR with the same 10 datasets.

### Statistical analysis

Statistical analysis was performed with SPSS (version 22, International Business Machines, Armonk, New York, USA). Data were tested for normality using the D’Agostino and Pearson omnibus normality test and values are presented as mean ± standard deviations, unless otherwise specified. For 2 group comparisons the Student’s t test was used for normally distributed data or Mann Whitney U test was used for non-normally distributed data. Repeated measures ANOVA with post hoc Tukeys’s multiple comparisons test or Friedman test with post hoc Dunn’s multiple comparison tests were performed for normally and non-normally distributed multiple groups respectively. *P* values < 0.05 were considered significant. Correlation was assessed using the Pearson or Spearman method as appropriate. Repeatability was assessed by consideration of the absolute difference between the results obtained from scan 1 and 2 for each subject. The coefficient of variance (CoV) was calculated for each subject, using the root mean square method [13]. The average CoV for a group for scan-rescan and interval scan repeatability was calculated by summing the squares of the variance for each subject, then taking the mean of the CoVs for all subjects and then square rooting this value. Mann Whitney tests were conducted to compare the CoV for the scan-rescan results to the CoV for the interval scan results. Bland-Altman plots [14] were used to display the differences between the paired datasets.

## Results

### Participant characteristics

Forty-five participants were recruited; mean age 54 ± 14 years (range 24–75 years) and 27 (59%) male. Demographic, anthropometric and routine CMR measurements are shown in Table 1. All LV volumes were within the normal range [15] and the mean ejection fraction was 66 ± 4%.

**Table 1:** Demographic, anthropometric and routine CMR measurements

	Controls *N* = 45
Age, years	54 ± 14 (range 24–75)
Male, %	59
Systolic blood pressure, mmHg	133 ± 20
Diastolic blood pressure, mmHg	78 ± 10
BMI, kg/m^2^	24.7 ± 3.8
Heart rate, beats per minute	65 ± 13
CMR data	
LV ejection fraction, %	66 ± 4
LV end diastolic volume, ml	155 ± 31
LV EDV indexed BSA, ml/m^2^	82 ± 14
LV end systolic volume, ml	52 ± 13
LV stroke volume, ml	103 ± 21
Cardiac output, L/min	6.5 ± 1.4

Values are mean ± standard deviations

*BMI* indicates Body mass index, *CMR* Cardiovascular magnetic resonance, *LV* Left ventricle, *EDV* End diastolic volume, *BS*A Body surface area

### 4D flow components’ volume and kinetic energy

All 80 data acquisitions passed data quality checks, with no significant difference in inflow versus ejected volume (inflow 82 ± 21 ml, outflow 81 ± 22 ml, *P* = 0.57).

Figure 1a demonstrates the visualised flow components, whilst Fig. 1b shows the average proportion of the 4 flow components as a percentage of the EDV for all 45 participants. The average contribution of each flow component, from largest to smallest, was: direct flow (38 ± 4%), residual volume (30 ± 4%), retained inflow (16 ± 4%) and delayed ejection flow (16 ± 3%). The results in Table 2 of repeated measures ANOVA comparisons with Tukey post-hoc testing demonstrate that the 4 flow components volumes were all significantly different to each other except for the retained inflow and delayed ejection flow.

**Table 2:** Multiple comparisons following repeated measures ANOVA for flow components as a percentage of EDV and Friedman test for KE at ED

	Mean ± SD	Retained inflow (*P* value)	Delayed ejection flow (P value)	Residual volume (P value)
Multiple comparisons for volume, % EDV				
Direct flow, % EDV	38 ± 4	< 0.001	< 0.001	< 0.001
Retained inflow, % EDV	16 ± 4	–	0.7534	< 0.001
Delayed ejection flow, % EDV	16 ± 3	0.7534	–	< 0.001
Residual volume, % EDV	30 ± 4	< 0.001	< 0.001	–
Multiple comparisons for KE at ED, μJ/ml				
Direct flow, μJ/ml	7.8 ± 3.0	< 0.001	0.0256	< 0.001
Retained inflow, μJ/ml	4.1 ± 2.0	–	0.0049	< 0.001
Delayed ejection flow, μJ/ml	6.3 ± 2.3	0.0049	–	< 0.001
Residual volume, μJ/ml	1.2 ± 0.5	< 0.001	< 0.001	–

*SD* indicates Standard deviation, *EDV* End-diastolic volume, *KE* Kinetic energy

The kinetic energy at ED in proportion to blood volume for each flow component is shown in Fig. 1c. The ED kinetic energy of each flow component, ordered from largest to smallest was: direct flow (7.8 ± 3.0 microJ/ml), delayed ejection flow (6.3 ± 2.3 microJ/ml), retained inflow (4.1 ± 2.0 microJ/ml) and residual volume (1.2 ± 0.5 microJ/ml). The mean KE at ED was statistically significantly different between all flow components, as found by comparison with Friedman test with post hoc Dunn’s testing demonstrated in Table 2.

### Intra and inter-observer variability

The results from the intra and inter-observer variability for the flow components as a percentage of the EDV are shown in Table 3. The coefficients of variation for the different flow components were low and similar for both intra and inter-observer results (range intra-observer 3.6–6.1%, vs inter-observer 2.6–5.7%), suggesting good intra and inter-observer variability.

**Table 3:** Intra and Inter-observer variability

	Intra-observer (Investigator 1)			Inter-observer (Investigator 2)	
	Analysis 1.1	Analysis 1.2	CoV	Analysis 2	CoV
Direct flow, % EDV	38 ± 4	37 ± 5	3.9	36 ± 5	5.7
Retained inflow, % EDV	14 ± 3	15 ± 3	3.6	15 ± 3	2.6
Delayed ejection flow, % EDV	17 ± 3	17 ± 3	6.1	18 ± 3	4.8
Residual volume, % EDV	31 ± 3	30 ± 3	3.5	31 ± 4	5.0
Ejection fraction, %	70 ± 3	69 ± 3	4.0	67 ± 4	4.1
EDV, ml	162 ± 46	166 ± 44	4.3	154 ± 40	5.8
ESV, ml	50 ± 16	51 ± 17	5.7	51 ± 16	5.6

*CoV* Coefficient of variation, *EDV* End diastolic volume, *ESV* End systolic volume

### Scan-rescan repeatability

**Fig. 2:**
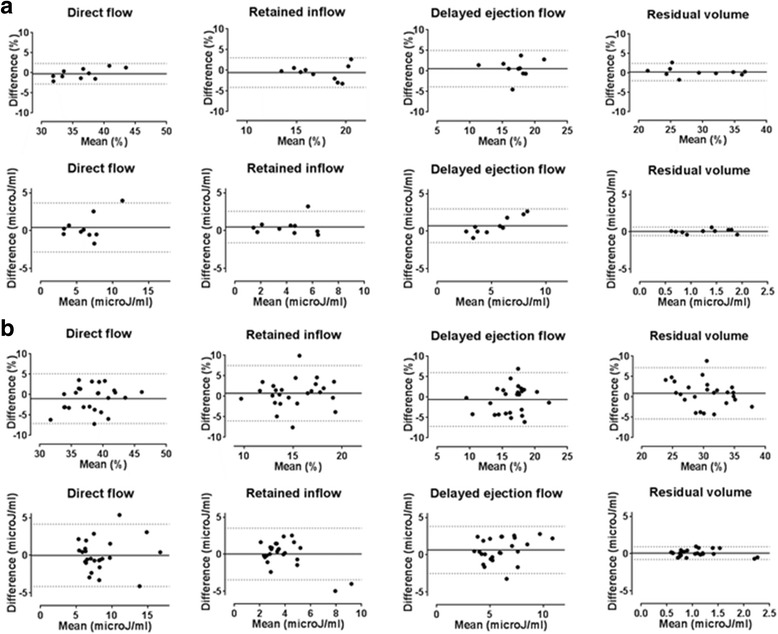
**a** Bland-Altman plots for scan-rescan data; flow components as percentage EDV (top row) and kinetic energy at end diastole (bottom row). Dotted lines represent 95% confidence intervals, unbroken line represents bias. **b** Bland-Altman plots for interval scan data; flow components as percentage EDV (top row) and kinetic energy at end diastole (bottom row). Dotted lines represent 95% confidence intervals, unbroken line represents bias

Ten subjects underwent 2 data acquisitions within the same study visit; Bland-Altman plots are shown in Fig. 2a for the relative volume and kinetic energy value at ED for each flow component. The observed mean difference values and coefficient of variation (CoV) for these results are summarised in Table 4. The flow component with the lowest CoV was the direct flow (2.5%), whilst the most variable flow component as a percentage of the EDV was the delayed ejection flow (CoV 9.2%). The kinetic energy values had higher coefficients of variation than those for the percentage of each flow component; with a range of 13.5% for direct flow to 17.7% for delayed ejection flow.

**Table 4:** Repeatability of measurements comparing scan-rescan and interval scans

	Scan-rescan		Interval scans		
	Observed mean difference (95% confidence interval)	CoV	Observed mean difference (95% confidence interval)	CoV	*P* value between CoV for scan-rescan and interval scans
End diastolic volume, ml	6.6 (−11.9 to 25.1)	4.6	1.1 (−19.2 to 21.4)	4.6	0.65
End systolic volume, ml	1.4 (−7.3 to 10.2)	6.8	−1.0 (− 13.3 to 11.3)	9.5	0.11
Left ventricular ejection fraction, %	0.4 (−5.5 to 6.5)	3.0	1.1 (−5.2 to 7.3)	3.3	0.12
Heart rate, bpm	4.3 (−6.9 to 15.5)	7.6	1.7 (−11.7 to 15.1)	6.8	0.18
Volume ratios					
Direct flow, % EDV	−0.2 (−2.8 to 2.3)	2.5	−1.0 (−7.1 to 5.1)	6.2	0.10
Retained inflow, % EDV	−0.5 (−4.1 to 3.0)	6.6	0.7 (−6.0 to 7.5)	16.1	0.04
Delayed ejection flow, % EDV	0.6 (−3.9 to 5.0)	9.2	−0.6 (−7.2 to 6.0)	15.0	0.07
Residual volume, % EDV	0.2 (−2.0 to 2.4)	3.0	0.9 (−5.4 to 7.2)	8.0	0.007
Kinetic energy					
Direct flow KE at ED, μJ/ml	0.4 (−2.8 to 3.7)	13.5	−0.01 (−4.2 to 4.2)	16.9	0.40
Retained inflow KE at ED, μJ/ml	0.5 (−1.6 to 2.6)	13.8	0.03 (−3.5 to 3.5)	20.4	0.13
Delayed ejection flow KE at ED, μJ/ml	0.7 (−1.5 to 3.0)	17.7	0.6 (−2.5 to 3.8)	27.5	0.16
Residual volume KE at ED, μJ/ml	0.06 (−0.5 to 0.6)	15.4	0.1 (−0.8 to 0.9)	29.0	0.23

*CoV* Coefficient of variation, *EDV* End diastolic volume, *bpm* Beats per minute

### Interval scans: Evaluation of variability and physiological variability

**Fig. 3:**
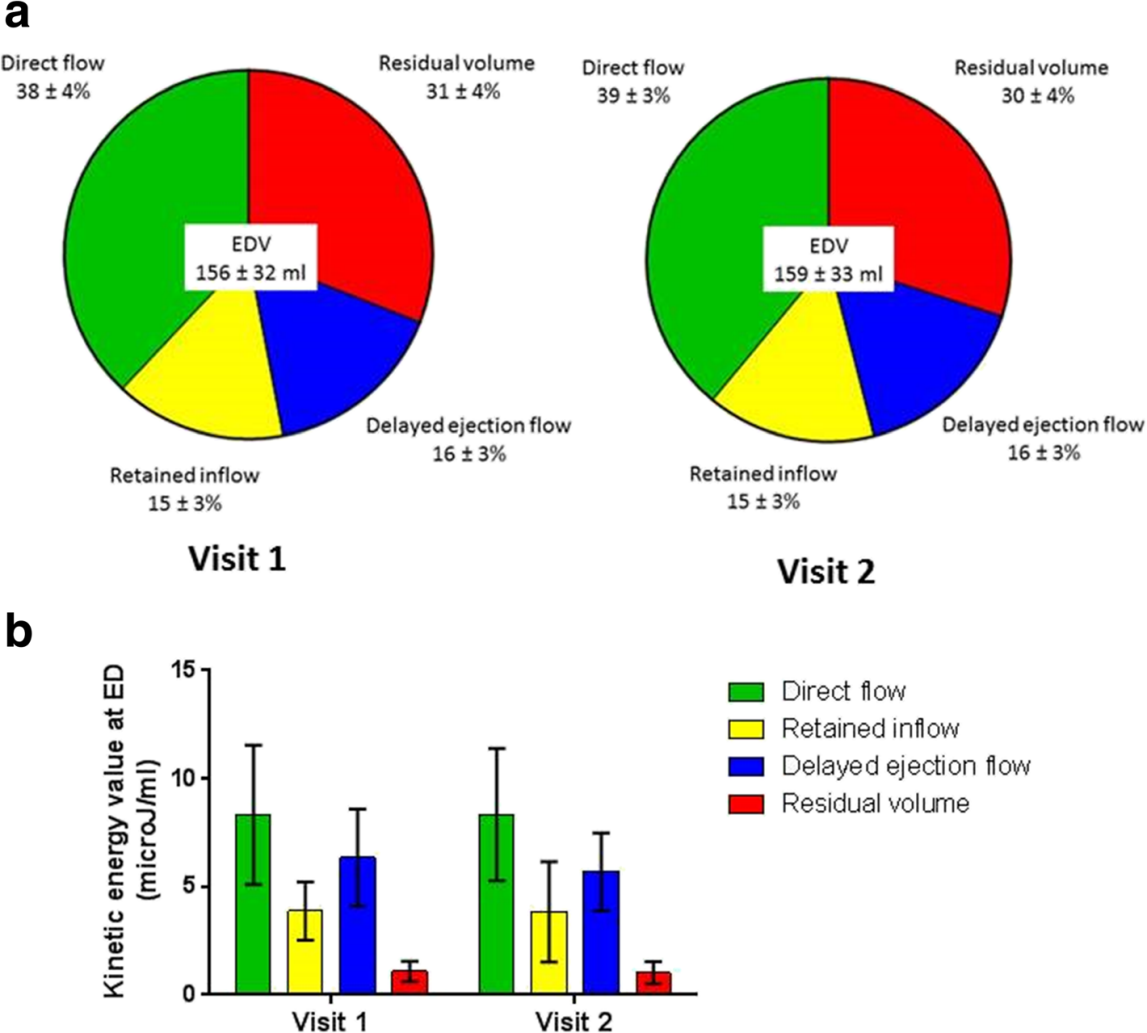
Interval scan popoulation differences in the 4 LV flow components for the 25 participants: **a** As percentage of EDV at visit 1 and 2. **b** Kinetic energy at end-diastole related to blood volume at visit 1 and 2

A total of 25 participants returned for a second scan after a median interval period of 52 days (IQR 28–57 days). The average proportion of each flow component as a percentage of the EDV did not differ significantly between visits, nor did the mean kinetic energy per millilitre at ED, as shown in Fig. 3. Figure 2b shows the Bland-Altman plots for the flow components’ volume and kinetic energy values; observed mean difference values and coefficients of variation are provided in Table 3. The 95% confidence intervals and CoV for each parameter measured are increased compared to those for the 10 scan-rescan datasets. As before the most stable flow parameter by percentage of EDV was the direct flow (CoV 6.2%) and the most variable component was the retained inflow (CoV 16.1%). The coefficients of variation for the KE values were again higher than those for the flow components as a percentage of EDV. The least variable parameter was the direct flow kinetic energy (CoV 16.9%), whilst the residual volume was the most variable with a CoV of 29.0%.

In order to understand whether the variability seen in each flow component volume and associated KE value, between the interval scans, was related to physiological parameters the changes in these results were correlated against each other. The mean difference between scan 1 and 2 was calculated for heart rate (2 ± 7 beats/min), LV EDV (3 ± 11 ml), stroke volume (1 ± 9 ml), LV ejection fraction (EF) (1 ± 3%) and cardiac output (10 ± 90 ml). There were no correlations seen between any of the flow component percentages or KE changes with the change in stroke volume, LV EDV or cardiac output. The change in the KE of the delayed ejection flow correlated weakly to the change in EF (*r* = 0.435, *P* = 0.03). Correlations were seen between the change in heart rate and the breakdown of flow components as a percentage of the EDV (direct flow *r* = 0.509, *P* = 0.009, retained inflow *r* = 0.424, *P* = 0.035, delayed ejection flow *r* = 0.500, P = 0.009 and residual volume *r* = 0.414, *P* = 0.04).

## Discussion

This study presents results from the largest cohort to date of healthy subjects for the quantification of LV flow components’ volume and KE values. The baseline data are in agreement with those from previous studies [3, 5, 7] with direct flow being the largest flow component, as a percentage of the EDV. The direct flow is also the component that possesses the highest kinetic energy value at ED. We found the retained inflow and delayed ejection flow components to be very similar as a proportion of the EDV, which provides a reassuring data quality control as they are inter-related components. These components are inter-related as much of the incoming retained inflow is ejected during the subsequent cardiac cycle as the delayed ejection flow, furthermore the volumes must be the same to preserve the cardiac volume. We successfully conducted scan-rescan data acquisitions to assess for repeatability and post-processing variability as well as interval scans to assess for the additional effect of physiological variability.

### Repeatability of data acquisition and post-processing

To our knowledge this is the first study to report 4D flow data from scan-rescan data acquisitions, where the aim was to attempt to quantify the magnitude of variation that is due to inherent sources of error in the data acquisition, post-processing and analysis. The scan-rescan repeatability was good for the flow components as a proportion of the EDV and slightly more variable for the KE values. The coefficients of variation we found for our flow component volumes were of a similar magnitude to those found in LV volumes calculated by cine CMR for conventional LV parameters including LV EDV, LV ESV and LV EF that found coefficients of variation between 4.1–10.3% [16].

Analysis of the influence of the post-processing steps including LV segmentation and selection of the appropriate time frame for ED and ES was assessed via the intra and inter-observer variability. The coefficients of variation for the flow component proportions were very similar between the intra and inter-observer variability results. These results are in agreement with previous studies assessing intra and inter-observer variability for cine CMR LV volumes [16, 17] and a study assessing flow component proportions that showed no difference in the group means and standard deviation [3]. These findings are expected; the main influence the operator has upon the post processing of the data is the contouring of the short axis ventricular images at ED and ES, which is then used to create the mask for analysis of the flow data. Both investigators undertaking analysis in this study are experienced at placing LV contours and were trained in the same CMR unit so will have similar contouring styles. The intra-observer coefficient of variation for the flow components as a proportion of the EDV was similar to that obtained for the scan-rescan data analysis suggesting that much of the variability in the results will be from the data analysis steps and not the data acquisition itself.

### Influence of physiological variability upon interval scans

The flow components as a percentage of the EDV and the KE of each component at ED were not significantly different between visit 1 and 2 across the group, however on a per participant basis there was greater variability between visit 1 and 2.. As expected the coefficients of variation were higher for all measured parameters for the 2 scans performed at an interval compared to the 2 scans performed in the same study visit. The results from the scan-rescan data acquisitions provide an estimation of the variability in 4D flow data acquisition, post-processing and analysis. The additional variability seen with the interval scans over scan-rescan suggests that as well as the differences in data acquisition, post processing and analysis there are additional influencing factors. We hypothesised that the increased variability was most likely due to a degree of normal physiological variability. We found that the change in heart rate recorded between the 2 interval scans correlated modestly to the changes seen in the flow components as a proportion of the EDV. This is an interesting finding and may be explained by the longstanding physiological observation that changes in heart rate predominantly affect the diastolic phase of the cardiac cycle, as the systolic phase is of a relatively fixed duration [18]. Hence, it may be that as the heart rate changes the proportions of the flow components alter to adapt to the new length of the diastolic period, whilst still maintaining an efficient systolic ejection phase. This may be an important physiological adaptation for exercise, which would be interesting to assess in future studies, including in disease states. However, the correlations with heart rate alone were only modest, suggesting further additional physiological factors such as fluid status, vascular tone and hormonal influences may be implicated in the variability seen for interval data acquisitions.

### Individual flow components

Direct flow was consistently the largest component with the highest possession of energy at ED; it was also the least variable of the four flow components in both composition and KE values. The direct flow represents the blood that transits directly from the left atrium via the LV cavity to be ejected into the aorta within the same cardiac cycle. Previous studies have demonstrated that the direct flow follows an efficient pathway to the LV outflow tract with the shortest distance, more favourable angle and increased linear momentum in comparison to the other flow components [7]. This suggests that in a healthy heart the percentage of blood pumped via the efficient shorter pathway taken by the direct flow component is relatively consistent; this may allow conservation of the KE of this component, which may be important in reducing the additional energy that is required for its ejection during systole. The residual volume was the second most stable component over time in terms of composition, but possessed the most variable amount of KE. The residual volume is located at the periphery of the LV cavity, outlining the functional border of the chamber, providing a fluid-fluid interface that interacts with the exchanging blood flow [9]. The location and stability in terms of the percentage of the residual volume could imply it is a static component of the LV blood flow volume. However the variability of the KE it possesses suggests it remains part of the dynamic interactions that occur with the incoming and outgoing cardiac blood flow within the LV cavity during each cardiac cycle. This may be an important factor in preventing blood stasis and thrombus formation within the healthy heart.

The KE for the retained inflow component at ED was significantly lower than that of the delayed ejection flow. This finding is in keeping with the notion proposed by *Bolger* et al. [5] that the retained inflow blood has to decelerate at the end of diastole and then acquire additional kinetic energy prior to ejection during a subsequent systole (as part of the delayed ejection flow). Previous studies have demonstrated a late diastolic boost in the KE of the delayed ejection flow, which was presumed to result from transfer of energy from the inflow components, so it may be that the KE is interchanged predominantly from the retained inflow to the delayed ejection flow. The amount of energy present within the delayed ejection flow at ED was not significantly different to the energy possessed by the direct flow; suggesting that a threshold level of kinetic energy favours ejection of blood from the LV during systole.

### Clinical applications

The applicability of 4D flow to assess the severity of cardiac disease has been previously demonstrated in patients with clinically compensated mild heart failure secondary to dilated cardiomyopathy [8] and ischaemic cardiomyopathy [19]. The use of this technique in longitudinal clinical studies is now required to assess how these parameters change over time in patients with heart disease and whether 4D flow parameters provide additional information to current imaging techniques in monitoring these patients.

### Limitations

The 4D flow data acquisitions undertaken in this study were conducted at rest. Given the relationship between the change in heart rate and the flow components as a percentage of the EDV it would be interesting to assess whether these proportions vary to a greater degree in an exercising heart. Blood flow within the heart is a dynamic process and 4D flow data is acquired over many heart beats with the end data representing the blood flow over an averaged cardiac cycle. In order to understand further if haemodynamic changes during the data acquisition influenced the final results continuous monitoring of heart rate and central blood pressure would be needed. For these measurements to be reliable they would need to be invasive which we felt would be too high a burden for our participants. The findings presented here are from a single study site and although they are in keeping with previous studies, future studies comparing the same study participants at different sites would provide additional validation of the 4D flow LV flow components’ volume and KE profiles. The two study groups (scan-rescan versus interval scan) consisted of different participants. We cannot exclude an influence of this upon the results seen, but as both groups had normal cardiac function and similar flow parameters we would expect any variability to be the same between these two groups. Finally the participants enrolled in this study were all healthy subjects and the reproducibility of this technique in patients with cardiac disease remains to be investigated. However it is unlikely that this technique will have less variability over time in patients with heart disease than controls, so this data may still act as an aid in assessing the significance of any changes in 4D flow parameters seen with longitudinal studies.

## Conclusions

This study provides an increased understanding of the variability of blood flow within the healthy heart. LV flow components’ relative volume and kinetic energy values are repeatable and are stable within a population over time. However, the variability of these measures in individuals over time is greater than can be attributed to inherent sources of error in the data acquisition, post processing and analysis, suggesting that additional physiological factors may influence the volume and KE profiles of the flow components. The assessment of intra-cardiac blood flow may become helpful in examining disease states and quantification of the variability of the results from this technique prior to this use is important.

## Additional file

Additional file 1: Table S1. CMR data for scan-rescan results compared to interval scan results. (DOCX 11 kb)

## Abbreviations

4D flow: Four dimensional flow
BMI: Body mass index
bSSFP: balanced steady-state free precession
CMR: Cardiovascular magnetic resonance
CoV: Coefficient of variation
ECG: electrocardiogram
ED: End diastole
EDV: End diastolic volumes
ES: End systole
FoV: Field of view
KE: Kinetic energy
LV: Left ventricular

## References

[1] Richter Y, Edelman ER (2006). Cardiology is flow. Circulation.

[2] Calkoen EE, de Koning PJ, Blom NA, Kroft LJ, de Roos A, Wolterbeek R, Roest AA, Westenberg JJ (2015). Disturbed Intracardiac flow organization after Atrioventricular Septal defect correction as assessed with 4D flow magnetic resonance imaging and quantitative particle tracing. Investig Radiol.

[3] Eriksson J, Carlhall CJ, Dyverfeldt P, Engvall J, Bolger AF, Ebbers T. Semi-automatic quantification of 4D left ventricular blood flow. J Cardiovasc Magn R. 2010;12. 10.1186/1532-429X-12-9 PMC2831022 20152026

[4] Wigstrom L, Ebbers T, Fyrenius A, Karlsson M, Engvall J, Wranne B, Bolger AF (1999). Particle trace visualization of intracardiac flow using time-resolved 3D phase contrast MRI. Magnet Reson Med.

[5] Bolger AF, Heiberg E, Karlsson M, Wigstrom L, Engvall J, Sigfridsson A, Ebbers T, Kvitting JPE, Carlhall CJ, Wranne B (2007). Transit of blood flow through the human left ventricle mapped by cardiovascular magnetic resonance. J Cardiovasc Magn R.

[6] Nilsson A, Bloch KM, Carlsson M, Heiberg E, Stahlberg F (2012). Variable velocity encoding in a three-dimensional, three-directional phase contrast sequence: evaluation in phantom and volunteers. J Magn Reson Imaging.

[7] Eriksson J, Dyverfeldt P, Engvall J, Bolger AF, Ebbers T, Carlhall CJ (2011). Quantification of presystolic blood flow organization and energetics in the human left ventricle. Am J Physiol-Heart C.

[8] Eriksson J, Bolger AF, Ebbers T, Carlhall CJ (2013). Four-dimensional blood flow-specific markers of LV dysfunction in dilated cardiomyopathy. Eur Heart J-Card Img.

[9] Carlhall CJ, Bolger A (2010). Passing strange flow in the failing ventricle. Circ-Heart Fail.

[10] Markl M, Bonk C, Klausmann D, Stalder AF, Frydrychowicz A, Hennig J, Beyersdorf F (2007). Three-dimensional magnetic resonance flow analysis in a ventricular assist device. J Thorac Cardiov Sur.

[11] Rider OJ, Lewandowski A, Nethononda R, Petersen SE, Francis JM, Pitcher A, Holloway CJ, Dass S, Banerjee R, Byrne JP (2013). Gender-specific differences in left ventricular remodelling in obesity: insights from cardiovascular magnetic resonance imaging. Eur Heart J.

[12] Heiberg E, Sjogren J, Ugander M, Carlsson M, Engblom H, Arheden H (2010). Design and validation of segment--freely available software for cardiovascular image analysis. BMC Med Imaging.

[13] Hyslop NP, White WH (2009). Estimating precision using duplicate measurements. J Air Waste Manag Assoc.

[14] Bland JM, Altman DG (1986). Statistical methods for assessing agreement between two methods of clinical measurement. Lancet.

[15] Petersen SE, Aung N, Sanghvi MM, Zemrak F, Fung K, Paiva JM, Francis JM, Khanji MY, Lukaschuk E, Lee AM, et al. Reference ranges for cardiac structure and function using cardiovascular magnetic resonance (CMR) in Caucasians from the UK biobank population cohort. J Cardiovasc Magn R. 2017;19. 10.1186/s12968-017-0327-9 PMC5304550 28178995

[16] Bogaert JG, Bosmans HT, Rademakers FE, Bellon EP, Herregods MC, Verschakelen JA, Van de Werf F, Marchal GJ (1995). Left ventricular quantification with breath-hold MR imaging: comparison with echocardiography. MAGMA.

[17] Hudsmith LE, Petersen SE, Francis JM, Robson MD, Neubauer S (2005). Normal human left and right ventricular and left atrial dimensions using steady state free precession magnetic resonance imaging. J Cardiovasc Magn R.

[18] Boudoulas H, Rittgers SE, Lewis RP, Leier CV, Weissler AM: Changes in diastolic time with various pharmacologic agents - implications for myocardial perfusion. Circulation 1978, 58(4):247-247. 10.1161/01.cir.60.1.164 376175

[19] Svalbring E, Fredriksson A, Eriksson J, Dyverfeldt P, Ebbers T, Bolger AF, Engvall J, Carlhall CJ (2016). Altered diastolic flow patterns and kinetic energy in subtle left ventricular remodeling and dysfunction detected by 4D flow MRI. PLoS One.

