# Supplementary material for: Test-retest variability of left ventricular 4D flow cardiovascular magnetic resonance measurements in healthy subjects
Source: J Cardiovasc Magn Reson. 2018 Mar 2;20:15. doi: 10.1186/s12968-018-0432-4 (PMC5833126; doi:10.1186/s12968-018-0432-4)
Supplement: Supplementary file 1 — CMR data for scan-rescan results compared to interval scan results. (DOCX 11 kb) [file 12968_2018_432_MOESM1_ESM.docx]

Additional file 1: Table S1

|  | Scan-Rescan  N=10 | Interval scan  N=25 | P value |
| --- | --- | --- | --- |
| **LV ejection fraction, %** | 64 ± 4 | 67 ± 4 | 0.10 |
| **LV end diastolic volume, ml** | 146 ± 37 | 156 ± 31 | 0.46 |
| **LV end systolic volume, ml** | 51 ± 12 | 52 ± 14 | 0.95 |
| **LV stroke volume, ml** | 95 ± 27 | 104 ± 20 | 0.28 |
| **Cardiac output, L/min** | 6.1 ± 1.4 | 6.6 ± 1.6 | 0.46 |
| **Direct flow, % EDV** | 37 ± 4 | 38 ± 4 | 0.36 |
| **Retained inflow, % EDV** | 17 ± 3 | 15 ± 3 | 0.10 |
| **Delayed ejection flow, % EDV** | 16 ± 3 | 16 ± 3 | 0.57 |
| **Residual volume, % EDV** | 30 ± 6 | 31 ± 4 | 0.27 |
| **Direct flow KE at ED, µJ/ml** | 6.5 ± 3.0 | 8.3 ± 3.2 | 0.12 |
| **Retained inflow KE at ED, µJ/ml** | 4.3 ± 2.0 | 3.9 ± 1.4 | 0.47 |
| **Delayed ejection flow KE at ED, µJ/ml** | 5.7 ± 2.4 | 6.3 ± 2.2 | 0.45 |
| **Residual volume KE at ED, µJ/ml** | 1.2 ± 0.5 | 1.1 ± 0.5 | 0.43 |
